# Educational Scoring System in Laparoscopic Cholecystectomy: Is It the Right Time to Standardize?

**DOI:** 10.3390/medicina59030446

**Published:** 2023-02-23

**Authors:** Elisa Reitano, Simone Famularo, Bernard Dallemagne, Kohei Mishima, Silvana Perretta, Pietro Riva, Pietro Addeo, Horacio J. Asbun, Claudius Conrad, Nicolas Demartines, David Fuks, Mariano Gimenez, Melissa E. Hogg, Charles Chung-Wei Lin, Jacques Marescaux, John B. Martinie, Riccardo Memeo, Olivier Soubrane, Michel Vix, Xiaoying Wang, Didier Mutter

**Affiliations:** 1 Research Institute against Digestive Cancer (IRCAD), 1 Place de l’Hôpital, 67000 Strasbourg, France; 2 Department of Translational Medicine, University of Eastern Piedmont, Via Solaroli 17, 28100 Novara, Italy; 3 Department of Digestive and Endocrine Surgery, University of Strasbourg, 67000 Strasbourg, France; 4 Hepato-Pancreato-Biliary Surgery and Liver Transplantation, Pôle des Pathologies Digestives, Hépatiques et de la Transplantation, Hôpital de Hautepierre-Hôpitaux Universitaires de Strasbourg, Université de Strasbourg, 1, Avenue Molière, 67098 Strasbourg, France; 5 Department of Surgery, Mayo Clinic, 4500 San Pablo Road, Jacksonville, FL 32224, USA; 6 Department of Surgery, St. Elizabeth’s Medical Center, School of Medicine, Boston University, Boston, MA 02135, USA; 7 Service de Chirurgie Viscérale, Département de Chirurgie, Centre Hospitalier Universitaire Vaudois, 1011 Lausanne, Switzerland; 8 Department of Digestive Surgery, Hôpital Cochin, 27 Rue du Faubourg Saint-Jacques, 75014 Paris, France; 9 Institut Hospitalo-Universitaire-Strasbourg (IHU-Strasbourg), 67200 Strasbourg, France; 10 Department of Surgery, University of Pittsburgh Medical Center, Pittsburgh, PA 15260, USA; 11 Show Chwan Memorial Hospital, Changhua 505, Taiwan; 12 Department of Surgery, Carolinas Medical Center, 1025 Morehead Medical Drive, Suite 600, Charlotte, NC 28204, USA; 13 Hepato-Pancreato-Biliary Surgery Unit, Miulli Hospital, Acquaviva delle Fonti, 70124 Bari, Italy; 14 Institut Mutualiste Montsouris, Université Paris Descartes, 75014 Paris, France; 15 Department of Liver Surgery and Transplantation, Liver Cancer Institute, Zhongshan Hospital, Fudan University, Shanghai 200433, China

**Keywords:** surgical education, cholecystectomy, learning curve

## Abstract

*Background and Objectives*: Laparoscopic cholecystectomy (LC) is one of the most performed surgeries worldwide. Procedure difficulty and patient outcomes depend on several factors which are not considered in the current literature, including the learning curve, generating confusing and subjective results. This study aims to create a scoring system to calculate the learning curve of LC based on hepatobiliopancreatic (HPB) experts’ opinions during an educational course. *Materials and Methods*: A questionnaire was submitted to the panel of experts attending the HPB course at Research Institute against Digestive Cancer-IRCAD (Strasbourg, France) from 27–29 October 2022. Experts scored the proposed variables according to their degree of importance in the learning curve using a Likert scale from 1 (not useful) to 5 (very useful). Variables were included in the composite scoring system only if more than 75% of experts ranked its relevance in the learning curve assessment ≥4. A positive or negative value was assigned to each variable based on its effect on the learning curve. *Results*: Fifteen experts from six different countries attended the IRCAD HPB course and filled out the questionnaire. Ten variables were finally included in the learning curve scoring system (i.e., patient body weight/BMI, patient previous open surgery, emergency setting, increased inflammatory levels, presence of anatomical bile duct variation(s), and appropriate critical view of safety (CVS) identification), which were all assigned positive values. Minor or major intraoperative injuries to the biliary tract, development of postoperative complications related to biliary injuries, and mortality were assigned negative values. *Conclusions*: This is the first scoring system on the learning curve of LC based on variables selected through the experts’ opinions. Although the score needs to be validated through future studies, it could be a useful tool to assess its efficacy within educational programs and surgical courses.

## 1. Introduction

Laparoscopic cholecystectomy (LC) is one of the most widely performed surgical procedures worldwide [1]. LC was introduced in the early 1990s. In 1989, Professor Jacques Perissat, whose presentation was not accepted in the main program at the meeting of the Society of American Gastrointestinal Endoscopic Surgeons (SAGES) in Louisville, Kentucky, displayed a videotape on laparoscopic cholecystectomy describing the technique in a remote booth of the exhibition area. This videotape attracted a larger audience than the lecturers in the main auditorium, marking the beginning of the worldwide revolution in laparoscopic surgery [2].

LC is currently recognized as the gold standard for the treatment of symptomatic cholelithiasis [3]. From an educational standpoint, LC is considered a standard surgical procedure, and it is one of the first operations performed by surgeons during their training [1,4]. Indeed, like all surgical procedures, LC carries its risks, with a reported postoperative complication rate between 9 and 16% [5,6,7]. Bleeding and iatrogenic biliary injuries are the most common intraoperative and postoperative complications, often representing reasons for conversion and leading to an increased risk of mortality with a consequent longer length of hospital stay [8]. The incidence of biliary injuries associated with laparoscopy is 0.25 to 0.74% for “major injuries” affecting the common bile duct, the common hepatic duct, and the right hepatic branch as complete resection of biliary duct, whereas it is 0.28 to 1.70% for minor injuries which impact the cystic stump, the cystic duct, and the junction between the cystic duct and the main biliary duct [9].

The reported incidence of uncontrollable bleeding in LC can be up to 2% (reported range, 0.03% to 10%) [10].

As the risk of intraoperative and postoperative complications is mainly related to the characteristics of the patient and to the degree of gallbladder inflammation, the Tokyo guidelines (TG18) provided recommendations on the surgical and clinical approaches to be adopted in emergency settings according to the grade of cholecystitis [11]. Consequently, the risk of complications is lower in elective settings and becomes greater as inflammation increases [8]. To perform a safe cholecystectomy, a thorough knowledge of normal biliary anatomy and its related variations, the identification of predictive factors for difficult surgery, as well as the use of correct techniques are considered fundamental [3]. Different articles have attempted to assess the learning curve for this procedure [12,13,14]. However, the available literature has yet to clearly determine the variables to be considered in measuring the learning curve, with large differences among studies [13,15] that do not make it possible to draw any solid conclusions [16]. The characteristics of the gallbladder and the patient, as well as the degree of inflammation, are taken into account by only few articles currently [16]. Consequently, the learning curve is often calculated from procedures where patient characteristics and the degree of complexity vary greatly. A recent systematic review [16] confirmed this heterogeneity. Indeed, the parameters to calculate the learning curve are not standardized and different authors considered different variables to evaluate the proficiency of surgical skills. The difficulty of the clinical setting and the risk of complications related to it are often not considered. The definition of the learning curve itself may be not very objective and is prevalently based on arbitrarily selected parameters. Surgical education is an active field of research, with increasing relevance especially in the field of minimally invasive surgery [4]. It seems crucial to define more objective and reproducible criteria to evaluate the surgical learning curve of such a widely performed intervention as laparoscopic cholecystectomy [16]. The aim of this study is to determine and define the variables that should be considered in the learning curve of LC and create a scoring system for learning curve assessment based on experts’ opinions during a dedicated surgical course. To our knowledge, this is the first study that has proposed implementing an educational scoring system using this methodological approach.

## 2. Materials and Methods

A short questionnaire was submitted to the panel of experts attending the hepatobiliopancreatic (HPB) surgery course at the Research Institute against Digestive Cancer (IRCAD, Strasbourg, France) from 27–29 October 2022. The selected experts were invited to participate in the course independently and were unrelated to the execution of this study. 

Experts who attended the course via Zoom videoconferencing filled out the questionnaire through a dedicated link. 

A list of 25 variables was submitted to the expert panel. Participants were asked to rank each variable’s impact on the LC learning curve, which enabled the authors to determine which ones should be taken into consideration to assess a surgeon’s progress along the learning curve. 

The variables submitted to the experts were extracted from the review of the current available literature on the learning curve of LC [16]. Learning curve variables were divided into 3 groups (preoperative, intraoperative, postoperative) and were ranked by each expert using a Likert scale from 1 (not useful) to 5 (very useful). According to the recent literature, minor injuries of the biliary tract were defined as injuries caused by electrocautery burns or a partial cut from sharp dissection with shears and not associated with tissue loss. Major biliary injuries were associated with tissue loss (e.g., clipping and transection of the common bile duct), hence requiring complex reconstruction with a Roux-en-Y hepaticojejunostomy [17]. Intraoperative complications were defined as events occurring from the first incision up until port removal [18], whereas postoperative complications were defined as events occurring after port removal in relation to the performed surgery [19]. 

Variables were included in the composite scoring system only if more than 75% of experts ranked its relevance in learning curve assessment as ≥4 (useful or very useful). This value was selected in accordance with the Delphi recommendations on how to report the ranking and scoring of medical educational research [20,21]. Each selected variable was ranked according to the mean score achieved on the Likert scale. In other words, the mean score that a variable obtained from the expert evaluations represents the value (rounded up) of the variable in the learning curve scoring system. 

Two variables (BMI and degree of inflammation) were divided into subcategories according to the literature, as the occurrence of different degrees of inflammation or the different classes of patient BMI would affect the difficulty of the surgical intervention. 

In this case, the mean score obtained from the expert assessments represented the maximum or minimum learning curve score of the possible subcategories. As a result, the BMI was divided into 5 grades [22], and each grade was assigned an increasing degree of difficulty up to a maximum of 4. The grade of inflammation [11] was divided into 3 classes according to the recent guidelines (grade I-II-III), and scores of 5 and 4 were assigned to stages II and I, respectively. As the Tokyo guidelines [11] suggested that grade III cholecystectomy should be performed laparoscopically only by experienced surgeons who have completed their learning curve, this grade was excluded from our scoring system. 

A positive or negative score was assigned to each variable according to its effect on the learning process. 

The scoring categories were compiled in accordance with the recent studies [11,22]. 

Cronbach’s alpha coefficient was calculated to determine overall consistency among experts (a value of ≥0.7 was considered an acceptable agreement). 

Data were recorded in a computerized Excel spreadsheet (Microsoft Excel 2016; Microsoft Corporation, Redmond, WA, USA) and analyzed with statistical software (IBM Corp. Released 2012. IBM SPSS Statistics for Windows, Version 21.0. Armonk, NY, USA: IBM Corp.).

## 3. Results

Fifteen experts (D.M.; M.H.; H.A.; M.G.; C.C.; X.W.; M.V; D.F.; N.D.; R.M.; J.M.; C.L.; P.A.; O.S.; J.M.) from six different countries (Argentina, France, Italy, Japan, Switzerland, and USA) attended the IRCAD HPB course and filled out the questionnaire. Table 1 outlines the question-and-answer (Q&A) options submitted to the experts and the relative scores on the definition of the learning curve of LC.

According to the most accepted answer (66.7% of preferences), the proposed definition of the learning curve was “The time taken and/or the number of procedures an average surgeon needs in order to be able to perform a procedure independently without intraoperative and postoperative complications”. As many factors could well influence the learning curve in the clinical practice, Table 2 outlines the different variables and their corresponding ranking using the Likert scale.

**Figure 1:**
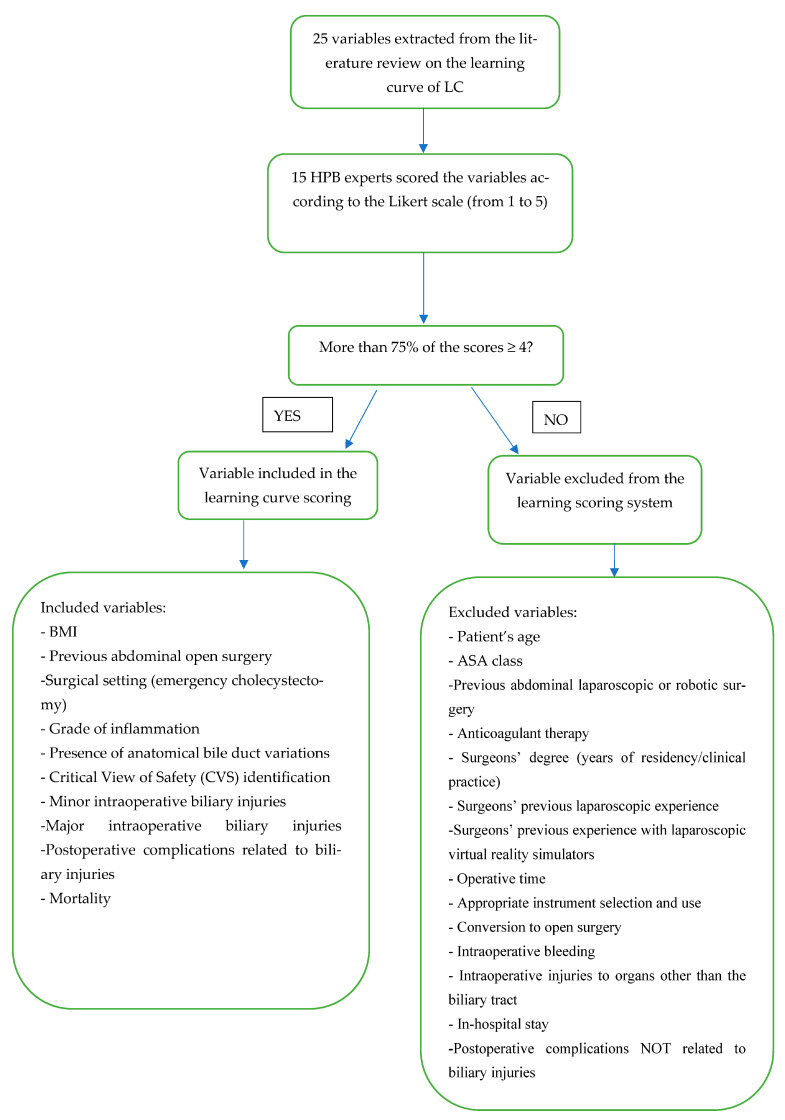
Flowchart of the selection process. LC: laparoscopic cholecystectomy; HPB: hepatobiliopancreatic; BMI: body mass index; BT: biliary tract.

According to the experts’ ranking, 10 variables were included in the final learning curve scoring system (Table 3), as more than 75% of the experts considered these variables to have an impact on the learning process (20). Figure 1 outlines the flowchart of a variable’s selection process.

Concordance between the examiners in ranking the different variables was acceptable with a Cronbach’s alpha coefficient of 0.756. After scoring each variable according to the mean score achieved on the Likert scale, a positive or negative score was assigned based on its effect in the learning process. To clarify the concept, as “patient previous open surgery” obtained a mean rate of 4.27 at the expert’s assessment, the value assigned to this variable within the scoring system was positive (+4). BMI, patient previous open surgery, emergency setting, increased levels of inflammation, presence of anatomical bile duct variations, and appropriate critical view of safety (CVS) identification were assigned positive values since they made surgery more difficult or represented pivotal points for patient safety. Major and minor intraoperative injuries to the biliary tract, development of postoperative complications related to biliary injuries, and mortality were assigned negative values as their presence impacted the success of the surgery. Table 3 outlined the definitive scoring system starting from the average marks received by the experts. 

According to the present scoring system, the maximum possible score was 27 (achievement of all positive scores while avoiding negative ones) and the minimum possible score was −17 (achievement of all negative scores with no positive score at all).

## 4. Discussion

The proposed scoring system represents the first attempts to quantify the learning process of LC in a reproducible way based on expert consensus regarding the type and relevance of the variables taken into consideration. Learning curves are particularly valuable in surgery to prevent operative mortality and morbidity, with major consequences on patient satisfaction and postoperative quality of life [6]. The issue of patient safety was highlighted by the UK General Medical Council Enquiry into the Bristol Pediatric Surgical Unit where concerns were raised about patients being exposed to surgeons in the early phase of the learning curve [23]. Guidelines are considered fundamental in medicine, representing key recommendations for the diagnosis and management of different procedures and diseases [24]. Currently, different guidelines and scoring systems orientate surgical decision-making, especially in complicated settings, often proposing alternative techniques such as fundus-first cholecystectomy, subtotal one, or conversion to open surgery [25]. Despite the growing value of surgical education, recommendations and guidelines are lacking in this field of research. Studies on surgical education are often performed in a subjective and non-reproducible manner, resulting in very different and confusing findings [16]. Poor reproducibility and integrity of the study may lead to ineffective interventions and poor clinical applications [26]. Indeed, the reproducibility of studies and experiments is particularly crucial in medicine as it serves as evidence that an established and documented study can be verified, repeated, and reproduced [26]. This concept applies to both the clinical settings and the educational fields, as educational courses and programs in medicine should be tailored and designed according to the evidence of an effect in skill development [27]. 

Standardizing the learning curve assessment in surgery seems fundamental to understanding the improvement in learning over time and with surgical experience in order to prevent surgical complications and improve patient outcomes. As there are no clear guidelines for calculating the learning curve in clinical practice [16], a clear educational scoring system could help obtain more reliable and reproducible findings in relation to assessments of the learning curve. Our educational scoring system could be particularly relevant in residency programs or in clinical fellowships to quantify student improvement in this specific procedure over time. Several authors have tried to assess the improvement in surgical skills during dedicated educational programs [28,29]. The results of these studies are often very subjective, based on different hypotheses, and not reproducible in other centers. With our own scoring system, each individual procedure could be evaluated, providing residents and surgeons with an immediate evaluation of their activity. The derived curves that could be obtained by plotting the scores over time can be useful for residency program directors to evaluate the residents’ yearly improvements in their practice of LC. As a result, the different points obtained for each variable could also be used to highlight the curve of growth. Another application of this study could be to identify which should be the endpoints selected to determine a learning curve (by the application of CUmulative SUM control chart—CUSUM or Risk-Adjusted Cumulative SUM—RACUSUM analyses) [30,31]. These are currently the most used methods to calculate the learning curve in medicine in reference to time (CUSUM) and other factors that might influence learning (RACUSUM) [32,33]. Consequently, beyond an immediate feedback based on the individual score, our scoring system can also assess learning progress over time. As previously stated, the variables submitted to the experts were extrapolated from the current literature and constitute the last literature review on the topic [16]. Voitk A. [34] estimated a learning curve of 200 LCs only taking into account operative time, complications, conversions, and readmission rates, while Moore et al. [35] set the learning curve at 50 cholecystectomies only considering the occurrence of bile duct injuries. Only three studies [13,15,36] considered the preoperative data, and only one author calculated a learning curve fixing it at 20 cholecystectomies [36]. Five studies [12,13,15,36,37] considered the expertise of the operator in assessing the learning curve and six studies [12,13,15,34,36,37] considered operative time, which was not considered by our experts as fundamental to the learning process of LC (Table 2). More than half of the experts (53.3%) rated operative time as not critical in assessing surgical progress; more variables related to patient safety (such as CVS identification and prevention of biliary injuries) were considered more important in learning curve assessment. This highlights the importance of having a learning curve based on patient outcomes and not only on operative time. Indeed, several studies on other surgical procedures showed that a shorter operative time is not always correlated with better clinical outcomes [38,39]. With respect to the available literature, some references to international definitions have been included to standardize the results. The Tokyo guidelines [40] were chosen to standardize the grade of difficulty of emergency LC. The latest version of the Tokyo guidelines (TG18) [11] available at the time of the draft of this article was chosen to categorize the different levels of surgical complexity according to the degree of gallbladder inflammation. The CVS was chosen to assess the correct recognition of the critical anatomical structures as different studies [41,42] demonstrated their educational value and their role in preventing minor and major complications. Indeed, before the introduction of laparoscopy, the “infundibular” technique, and the intraoperative recognition of the cystic duct and gallbladder junction for gallbladder hilar dissection were the preferred surgical methods [3]. In 1995, Strasberg introduced the concept of the “Critical View of Safety” (CVS) to promote the identification of the gallbladder structures in order to reduce the risk of biliary injuries originating from anatomical alterations and altered visual perception [3].

Recently, SAGES recognized the importance of CVS identification, encouraging the use of this technique in the “Safe Cholecystectomy Program” to minimize the risk of biliary injuries [43]. Finally, it should be noted that most of the experts rated previous experience with virtual reality simulation as “not very useful”, and as a result, this was excluded from our scoring system. Simulation is gaining increasing popularity in surgical training [40]. It is commonly accepted that virtual reality simulators allow surgeons to decrease the learning curve for complex surgical skills in a controlled environment without jeopardizing patient safety [44]. Although different studies [45,46] suggest that virtual reality simulators play a role in surgical skills progression, the experts in our study ranked previous experience with these instruments as a less impactful criterion on which to base learning curve calculation. Indeed, some studies underlined the low level of validity of some simulators and the difficulty of skills transfer to the real environment [44].

Cirocchi et al. showed that in many cases of malpractice claims and civil action suits, the patients’ morbidity and mortality were related to a misperception of the CVS, and not due to the surgeon’s negligence [47]. In such cases, stopping the procedure, taking time to reorientate the anatomical landmarks, or asking for help from a more experienced colleague would be the best options available [47]. Once again, factors related to patient safety and outcomes were considered fundamental compared to previous studies. 

Our scoring system has a maximum score of 27 and a minimum of −17. In clinical practice, it is difficult to reach such extreme scores. The score of 27 should be reflected in the case of a patient with a BMI > 40, who had had previous open surgery, and who was operated on for an emergency grade II cholecystitis with anatomical biliary tract variations, in which the CVS had been correctly identified and without any intraoperative and postoperative complications related to biliary injuries and no mortality. The minimum score system refers to a patient with normal body weight, without previous open surgery, operated on in an elective setting without any anatomical biliary tract variations, in whom the CVS is not correctly identified, with minor and major intraoperative biliary injuries, and with the postoperative development of biliary-related complications with the patient’s death. The upper cut-off value should subsequently not be intended as a value to achieve the learning curve. Achieving higher scores over time or maintaining positive values would help easily quantify an improvement in surgical skills. 

However, this study has several limitations. Although the selection and the ranking of variables were made in accordance with the experts’ opinions, this does not represent an official guideline or consensus. The number of experts was limited to those attending the IRCAD HPB course and the results would differ with a larger sample size. However, as all experts were leading or involved in educational programs, we thought that the present score could have a scientific and educational value. This study also emphasized the need for the surgical training to be more objective and reproducible. Many variables usually considered critical in the learning curve process within the current literature were deemed not so fundamental by our panel of experts (for example, operative time). Finally, the validation of this score and its usefulness will have to be tested in clinical practice with further studies required within educational programs. After validation, a dedicated online calculator will be developed to facilitate learning curve assessment.

## 5. Conclusions

Our educational scoring system could be a useful tool to assess improvements in surgical skills and in the learning curve of LC, one of the most performed surgeries across the globe. This scoring system could be particularly helpful in educational and residency programs. Compared to the CUSUM and RACUSUM analyses, it represents a very intuitive method to calculate the learning curve, which can also provide a direct idea of the learning process without necessarily resorting to complex statistical analyses. The scoring system could also be useful to identify the endpoints to be considered when determining a completed learning curve.

Further studies are necessary to validate the scoring system within dedicated educational programs.

## Figures and Tables

**Table 1:** Definition of learning curve.

A Surgical Learning Curve Is Defined as “The Time Taken and/or the Number of Procedures an Average Surgeon Needs in Order to Be Able to Perform a Procedure Independently with a Reasonable Outcome”. With Respect to the Learning Curve of LC, What Is a Reasonable Outcome in Your Opinion?	Nb (%) of Experts Who Chose the Corresponding Definitions
(1) Completing the surgery with no intraoperative complications.	3 (20)
(2) Completing the surgery as quickly as possible (short operative time).	1 (6.7)
(3) Having no early postoperative complications.	0
(4) Having no early or long-term postoperative complications.	1 (6.7)
(5) Having no intraoperative or postoperative complications.	10 (66.7)

Table 1: LC: laparoscopic cholecystectomy.

**Table 2:** Variables ranked by the experts.

Variables	Likert Score 1–3 [N (%)]	Likert Score 4≥ [N (%)]	To Be Included in the Scoring System (Y/N)
Preoperative factors			
Patient’s age	15 (100)	-	N
BMI	-	15 (100)	Y
ASA class	13 (86.7)	2 (13.3)	N
Previous laparoscopic or robotic abdominal surgery	11 (73.3)	4 (26.7)	N
Previous open abdominal surgery	2 (13.3)	13 (86.7)	Y
Anticoagulant therapy	12 (80)	3 (20)	N
Surgery setting (elective or emergency cholecystectomy)	-	15 (100)	Y
Grade of inflammation according to Tokyo Guidelines (in emergency cholecystectomy)	-	15 (100)	Y
Surgeons’ degree (years of residency/clinical practice)	7 (46.7)	8 (53.3)	N
Surgeons’ previous laparoscopic experience (other than cholecystectomy)	4 (26.7)	11 (73.3)	N
Surgeons’ previous experience with laparoscopic virtual reality simulators	11 (73.3)	4 (26.7)	N
Presence of anatomical bile duct variations	2 (13.3)	13 (86.7)	Y
Intraoperative factors			
Operative time (from the first incision to port removal)	8 (53.3)	7 (46.7)	N
Critical view of safety (CVS) identification	-	15 (100)	Y
Appropriate instrument selection and use	6 (40)	9 (60)	N
Conversion to open surgery	7 (46.7)	8 (53.3)	N
Intraoperative bleeding	6 (40)	9 (60)	N
Minor intraoperative injuries to the BT	2 (13.3)	13 (86.7)	Y
Major intraoperative injuries to the BT	1 (6.7)	14 (93.3)	Y
Intraoperative injuries to organs other than the BT	4 (26.7)	11 (73.3)	N
Postoperative factors			
In-hospital stay	10 (66.7)	5 (33.3)	N
Postoperative complications related to biliary injuries	3 (20)	12 (80)	Y
Postoperative complications NOT related to biliary injuries	7 (46.7)	8 (53.3)	N
Mortality	2 (13.3)	13 (86.7)	Y
Readmissions	5 (33.3)	10 (66.7)	N

Table 2: BMI: body mass index; ASA: American Society of Anesthesiologists; CVS: critical view of safety; BT: biliary tree.

**Table 3:** Variables ratings and resulting scoring system.

Variables	Mean (±SD)	Score	Subcategory
Preoperative			
BMI	4.07 (0.799)	0 to +4	BMI 18–24.9: +0 BMI 25–29.9: +1 BMI 30–35: +2 BMI 35–40: +3 BMI > 40: +4
Previous abdominal open surgery	4.27 (0.884)	+4	
Surgical setting (emergency cholecystectomy)	4.60 (0.507)	+5	
Grade of inflammation according to Tokyo guidelines (in emergency cholecystectomy)	4.87 (0.352)	+4 or +5	Grade 1: +4 Grade 2: +5 Grade III excluded
Presence of anatomical bile duct variations	4.20 (1.08)	+4	
Intraoperative			
Critical view of safety (CVS) identification	4.67 (0.72)	+5	
Minor intraoperative injuries to the BT	4.07 (1.33)	−4	
Major intraoperative injuries to the BT	4.73 (1.03)	−5	
Postoperative			
Postoperative complications related to biliary injuries	4.07 (1.62)	−4	
Mortality	4.40 (1.40)	−4	

Table 3:BMI: body mass index; CVS: critical view of safety; BT: biliary tree; SD: standard deviation.

## Data Availability

Data are available upon request to corresponding author.
